# Meta-analysis of expression and the targeting of cell adhesion associated genes in nine cancer types – A one research lab re-evaluation

**DOI:** 10.1016/j.csbj.2023.04.017

**Published:** 2023-04-19

**Authors:** Olegs Borodins, Felix Broghammer, Michael Seifert, Nils Cordes

**Affiliations:** a OncoRay—National Center for Radiation Research in Oncology, Faculty of Medicine Carl Gustav Carus, Technische Universität Dresden, 01307 Dresden, Germany; b Institute for Medical Informatics and Biometry (IMB), Faculty of Medicine Carl Gustav Carus, Technische Universität Dresden, 01307 Dresden, Germany; c National Center for Tumor Diseases (NCT), Partner Site Dresden, German Cancer Research Center (DKFZ), 69192 Heidelberg, Germany; d Helmholtz-Zentrum Dresden—Rossendorf (HZDR), Institute of Radiooncology—OncoRay, 01328 Dresden, Germany; e German Cancer Consortium, Partner Site Dresden: German Cancer Research Center, 69120 Heidelberg, Germany; f Department of Radiotherapy and Radiation Oncology, University Hospital Carl Gustav Carus, Technische Universität Dresden, 01307 Dresden, Germany

**Keywords:** Integrins, Adhesion, Gene expression, Network analysis, Pancancer

## Abstract

Cancer presents as a highly heterogeneous disease with partly overlapping and partly distinct (epi)genetic characteristics. These characteristics determine inherent and acquired resistance, which need to be overcome for improving patient survival. In line with the global efforts in identifying druggable resistance factors, extensive preclinical research of the Cordes lab and others designated the cancer adhesome as a critical and general therapy resistance mechanism with multiple druggable cancer targets. In our study, we addressed pancancer cell adhesion mechanisms by connecting the preclinical datasets generated in the Cordes lab with publicly available transcriptomic and patient survival data. We identified similarly changed differentially expressed genes (scDEGs) in nine cancers and their corresponding cell models relative to normal tissues. Those scDEGs interconnected with 212 molecular targets from Cordes lab datasets generated during two decades of research on adhesome and radiobiology. Intriguingly, integrative analysis of adhesion associated scDEGs, TCGA patient survival and protein-protein network reconstruction revealed a set of overexpressed genes adversely affecting overall cancer patient survival and specifically the survival in radiotherapy-treated cohorts. This pancancer gene set includes key integrins (e.g. ITGA6, ITGB1, ITGB4) and their interconnectors (e.g. SPP1, TGFBI), affirming their critical role in the cancer adhesion resistome. In summary, this meta-analysis demonstrates the importance of the adhesome in general, and integrins together with their interconnectors in particular, as potentially conserved determinants and therapeutic targets in cancer.

## Highlights

• Preclinical studies support the relevance of adhesion mediated cancer resistance.
• Nine cancer types exhibit similar altered expression of adhesion associated genes.
• 41 adhesion associated genes show clinical impact on patient survival.

## Graphical Abstract

**Figure:**
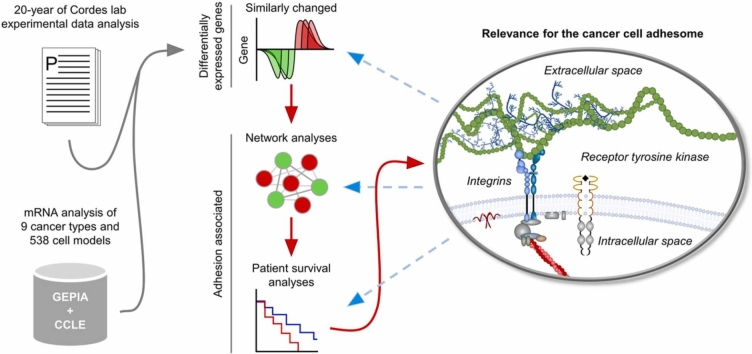

## 1 Introduction

The mortality rate of nearly ten million deaths worldwide in 2020 makes cancer a leading public health problem, and it is predicted to increase by about 50% within the next two decades [1]. Cancer is composed of a multitude of different diseases, all defined by malignant autonomous growth and spreading of somatic clones. Heterogeneity between and within cancer types is vast, presented by multi-layered disparities in morphology, (epi-)genetic changes and microenvironmental aspects [2], [3]. This diversity is also portrayed by the heterogeneous efficacy profiles of both conventional radio(chemo)therapies and novel molecular targeted treatment approaches [4]. Inherent and acquired resistance, resulting from genetic and epigenetic alterations, are the cause of low or absent treatment responses. As a consequence, signal transduction favoring survival as well as changes in the tumor microenvironment, such as hypoxia, induced angiogenesis and deposition of extracellular matrix (ECM) are found [5], [6], [7].

The role of the ECM receives increasing attention as cell-ECM interaction sites critically function in structural and regulatory processes. Cell-ECM interaction sites, called focal adhesions (FA), are formed by more than 200 adhesome proteins with 60 core regulators [8], [9]. FAs are intricate and dynamic structural nodes that connect the cytoskeleton and ECM, as well as form hubs of signal transduction that are simultaneously required for optimal regulation of numerous cellular functions such as adhesion, survival, proliferation, migration / invasion, and stemness [10], [11]. This multifunctionality is enabled by diverse sets of integrins, receptor tyrosine kinases, intracellular protein kinases and adapter proteins [10], [12]. Thus, in cancer, mechanical cues necessary for tissue remodeling and cell invasion as well as promitotic and prosurvival signaling are frequently hijacked to elicit the hallmarks of cancer [13], [14]. The redundancy of adhesome proteins and how they influence downstream signaling pathways remains variable and cancer type-specific, making conventional therapy and tailored approaches challenging [7]. However, numerous promising preclinical studies using various inhibitors against adhesome receptor and adapter proteins demonstrate the efficacy and feasibility of overcoming treatment resistance in head and neck cancer [15], prostate cancer [16], breast cancer [17], brain tumors [18], pancreatic cancer [19], lung cancer [20], skin cancer [21], hematopoietic system [22] and other cancers [7], [13].

The pioneering work of the Cordes lab on the cancer adhesion resistome uncovered shared traits in therapy resistance mechanisms across various cancer types. This paradigm indicates potentially conserved adhesion associated prosurvival signaling axes of general importance. To address this, the present meta-analysis took the published results of the Cordes lab over a period of 20 years to create a reasonable starting point for a connection with large cancer patient cohorts. Developments in next-generation sequencing technologies provide an unprecedented amount of publicly available cancer patient-specific omics data from large cooperative studies such as the Therapeutically Applicable Research to Generate Effective Treatments (TARGET) project [23] or The Cancer Genome Atlas (TCGA) [24]. By linking the expression and patient survival data of previously investigated cancer types with preclinical molecular targeting experiments on over 200 molecules, we demonstrated the reasonability and relevance of the body of work done in the Cordes lab. Specifically, we examined commonalities in adhesion associated genes identified critical for changes in therapy sensitivity in preclinical settings, placed them in a broader context, and explored their relevance to patient survival in general and with conventional radio(chemo)therapy in particular.

## 2 Materials and methods

### 2.1 Literature analysis

**Fig. 1:**
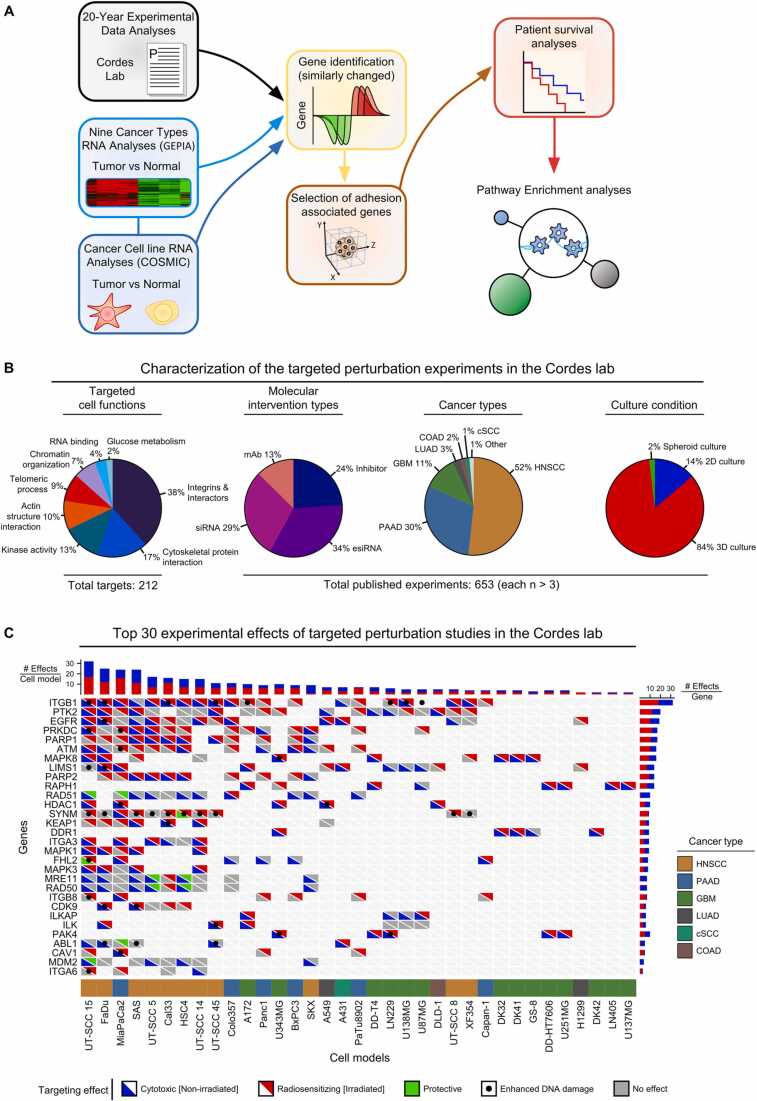
Workflow, experimental background and targeting effects in cell models from various cancer types. (A) Schematic of the study workflow. (B) Characterization of 653 published in vitro experiments (each consisting of a at least 3 biological replicates) performed in the Cordes lab. Differentiation was made between molecular intervention type, cancer type and cell culture condition. 212 unique targets were functionally classified by gene ontology analyses. (C) Top 30 targets affecting survival and DNA damage in 35 cell models from published Cordes lab experiments. Significant (p < 0.05) adverse effects on survival are designated as cytotoxic (blue; under non-irradiated conditions) and radiosensitizing (red; under irradiated conditions). A significant increase in survival is designated as protective (green). A targeting-mediated enhancement in DNA damage after irradiation is indicated by black dots. Gray color indicates no effects on cell survival. HNSCC, Head and neck squamous cell carcinoma; PAAD, Pancreatic adenocarcinoma; GBM, Glioblastoma multiforme; LUAD, Lung adenocarcinoma; cSCC, Cutaneous squamous cell carcinoma; COAD, Colon adenocarcinoma.

The publication body of the Cordes lab was examined between the years 2003 and 2022 for significant effects (p < 0.05) upon molecular intervention with single targets. The focus was put on the two relevant experimental endpoints clonogenic survival and DNA damage under nonirradiated and irradiated conditions. The collected effects of 212 targets in 51 normal and cancer cell models are shown in Fig. 1, Fig. S1 and Table S1. R (v4.2.1) was utilized for visualization (complex heatmap, v2.13.2). Additionally, an extensive PubMed literature search on similar endpoints was conducted on the 30 most investigated Cordes lab targets to gain a broad overview about the investigations by other research groups. For each target gene, original, free-full text articles published between 2003 and 2023 were considered fulfilling the following criteria: (1) the target gene plus its alias names had to be combined with one of the following terms: “Silencing”, “Knockdown”, “Inhibition”, “Inhibiting”, “Targeting”, “Perturbation”, or “Depletion”; (2) the articles had to contain one of the following terms: “Radiation”, “Radiosensitizing”, “Radioresistance”, “Radioprotection”, “Radiotherapy”, “Radiochemotherapy”, “Chemoradiation”; (3) the articles had to include the terms “Cancer” or “Tumor”; (4) the research had to be conducted in human cells (“humans[Filter]”). Two approaches were pursued with this search formula: (a) a detailed, strict search, where the exact search terms had to be located in the text of the article ([tw] after each term combination) and (b) a broader text mining approach, where the search terms were allowed to be present in any combination in the article. To further filter the broad search, we merely selected publications with a radiation associated title and excluded publications already addressed in the detailed, strict search (a). In total, we investigated 408 publications in the detailed, strict search (a) and 1123 studies in the broader text mining approach (b). The radiation-specific results are presented in Fig. S2. The complete literature search data is listed in Table S1.

### 2.2 RNA expression data

The GEPIA (Gene Expression Proﬁling Interactive Analysis) web tool (http://gepia.cancer-pku.cn/index.html) provides processed RNA sequencing data from 9736 cancers and 8587 normal tissues from multiple publicly available large cohort studies [25]. From GEPIA build-in ANOVA analysis, significant (q-value cutoff<0.05) differential expressed genes (DEGs) between 9 cancer types (Lung squamous cell carcinoma, LUSC; Lung adenocarcinoma, LUAD; Prostate adenocarcinoma, PRAD; Colon adenocarcinoma, COAD; Rectum adenocarcinoma, READ; Lower grade glioma, LGG; Glioblastoma multiforme, GBM; Head and neck squamous cell carcinoma, HNSCC; Pancreatic adenocarcinoma; PAAD) and corresponding normal tissues were acquired. Raw RNA sequencing data for different cell models were downloaded from the DepMap database (https://depmap.org/portal/) [26] and further normalized using a standard processing pipeline in R (DeSeq2, v1.38.0). The fold changes of the DEGs from implemented cell models were generated by comparison to tissue specific control datasets obtained from OncoBoxPD (https://open.oncobox.com) [27]. Coding and non-coding DEGs demonstrating similar expression changes found in at least 8 out of the 9 cancer types were named similarly changed DEGs (scDEGs) and considered for further analysis (Table S2).

### 2.3 Transcription factors

TFs were retrieved via analysis of the 9 overlapping cancer datasets with g:profiler [28] web tool (https://biit.cs.ut.ee/gprofiler/gost) and The Human Transcription Factors atlas v1.01 (http://humantfs.ccbr.utoronto.ca/) [29]. Identified TFs were used in the network analyses (Table S2).

### 2.4 Gene enrichment and network analyses

All enrichment and protein-protein interaction network analyses were conducted through the CytoScape platform (v3.4.0) [30]. The Cordes lab gene pool was enriched via GeneMANIA [31] and STRING [32] (75% confidence). By GeneMANIA, we reconstructed protein-protein interaction networks on the basis of physical interaction, co-expression, predicted colocalization, shared pathways, genetic interaction, and shared protein domains. The enriched dataset was overlapped with identified scDEGs and adhesion associated genes (defined by Gene Ontology (GO) [33]), and implemented into network analyses together with identified TFs. From the network analyses, all genes with 1st neighbor interaction to the initial Cordes lab dataset were retained. Only genes with a minimum of 2 degrees in the network and corresponding scDEGs with an average fold change of either> 1.5 or< 0.5 from normal tissue were considered for subsequent survival analysis and network reconstruction. We termed the 206 resulting genes adhesion associated scDEGs (Table S2). The gene set obtained from the survival analyses (aaGOIs-All/RT) was used to reconstruct the interactions via the GeneMANIA app as mentioned above. Prior to GeneMANIA reconstruction, genes were ordered based on high expression in cancer compared to the corresponding normal tissue.

### 2.5 Survival analysis

Survival and expression data of nine TCGA datasets (LUSC, LUAD, PRAD, COAD, READ, LGG, GBM, HNSCC, PAAD) were obtained from the Xena platform [34] and evaluated. The cancer type specific TCGA patient cohorts were divided for each analyzed gene into high and low expression cohorts by the group cutoff of median expression and subjected to individual Kaplan-Maier analysis. We selected overexpressed genes with positive or negative impact on patient overall survival (OS) after initial diagnosis for an explorative analysis, which were defined by p-value cutoffs (p < 0.1; p < 0.05; p < 0.01) from independent log-rank testing. From the complete TCGA cancer type cohorts (“All”), we further classified subcohorts of patients receiving radio(chemo)therapy only (“RT”) versus patients receiving no radiotherapy (“No RT”). The TCGA phenotypic identifiers “radiation therapy” and “additional radiation therapy” were used for differentiation of subcohorts. The “RT” and “No RT” subcohorts of COAD and READ were combined into one colorectal cancer cohort (COAD / READ) because of the small number of “RT” patients in the two cohorts (COAD: 20/394; READ: 24/139; source data provided in Table S3). Survival analyses were performed by R (survival v3.4.0; survminer v0.4.9).

### 2.6 Functional enrichment analysis

Functional pathway enrichment analysis of the scDEGs was performed via R (clusterProfiler v3.8) for GO and Kyoto Encyclopedia of Genes and Genomes (KEGG) [35]. The results were hierarchically clustered to classify the most suppressed and activated pathways. Adhesion associated scDEGs were additionally taken to reconstruct their 1st neighbor interaction between scDEGs. Functional analysis was performed on the identified gene set by OncoBoxPD to obtain equalized functional enrichment datasets. Genes identified after the survival analysis (aaGOIs-All/RT) were included in a pairwise clustering correlation evaluation of significant (p < 0.05; FDR<0.25) functional associations (1614 terms, acquired from Wikipathways, Reactome, KEGG, GO resources, Molecular Signatures Database (MSigDB); processed with g:profiler, ShinyGO, GSEA) [28], [33], [35], [36], [37], [38], [39], [40]. The clusters with a correlation coefficient of> 0.75 were considered and summarized (Table S4). Additionally, the same genes were integrated into a ClueGO [41] network analysis to identify an independent abundance of the GO terms. The aaGOIs-All/RT included in GeneMANIA network analysis were classified in ShinyGO according to GO molecular functions into the categories “Integrins”, “Integrin binding”, “Matrisome associated”, “Kinase”, “Cytoskeleton component”, “Signal transduction”, and “Transcription factors”.

### 2.7 Statistical analysis

The significance level of p < 0.05 and, where applicable, FDR< 0.05 was considered in this study if not stated otherwise. Pearson correlation analyses between different cell models and their corresponding cancer types were performed using the GraphPad Prism 7 software. Patient survival analyses for corresponding low- and high-expression patient cohorts were performed in R by log-rank testing for each gene in each cancer type independently with three p-value cutoffs (p < 0.1; p < 0.05; p < 0.01) to indicate the degree of significance. Pairwise correlation clustering (p < 0.05; FDR<0.25) between genes and functional terms was conducted by python spyder (matplotlib v3.6.2, SciPy v1.9.3) [42], [43].

## 3 Results

### 3.1 General workflow for dissecting common mechanisms in nine human cancer types in the context of molecular targeting approaches for adhesion associated molecules

The workflow in Fig. 1A indicates the various analytical steps in the presented meta-analysis. Firstly, we began to comprehensively collect experimental data on specific cancer targets in a radiobiological context from published Cordes lab studies between 2003 and 2022. During this period, the research group worked on adenocarcinoma of the lung (LUAD), adenocarcinoma of the prostate (PRAD), adenocarcinoma of the colon (COAD), glioblastoma (GBM), squamous cell carcinoma of the head and neck (HNSCC), and adenocarcinoma of the pancreas (PAAD). These are common cancers that exhibit a marked phenotypic and genetic heterogeneity. To gain a broader view on the relevance of this experimental body and to identify potential new routes for cancer resistome targeting, the extensively studied targets of the Cordes lab gene set were enriched with the closest gene interaction networks. Then, we aligned RNA sequencing datasets of cancer cell models with their corresponding cancer type datasets. Similarly changed differentially expressed genes (scDEGs) associated with cell adhesion were identified, aligned with the Cordes lab gene pool, and enriched for transcription factors (TF) and adhesion genes. From this gene pool, 1st neighbor gene interactions were reconstructed to the Cordes lab genes, which formed the adhesion associated scDEGs. To further elucidate the most relevant gene targets, systematic TCGA patient survival analyses were executed, followed by in-depth assembly of the interaction networks of radiotherapy- and poor prognosis associated genes to finally conclude with a functional enrichment analysis (Fig. 1A).

### 3.2 Cordes lab group literature analysis defines the study basis

The present study set out with a comprehensive data collection of the Cordes lab publication body, based on its broad expertise in the cancer cell adhesome and molecular targeting. Although numerous multi-targeting and chemotherapy-combination experiments were conducted over the years, the focus of this meta-analysis were single targeting experiments in order to obtain the distinct outcomes of individual molecule inhibitions. Collectively, a total of 212 targets were extensively evaluated in 653 published experiments, each with a minimum of 3 biological replicates (Fig. 1B). Responses of 51 cell models from 10 cancer types, immortalized fibroblasts and keratinocyte cultures towards small molecule inhibitors, inhibitory antibodies or RNA interference (RNAi)-mediated targeting approaches were recorded. Notably, in over 85% of experiments, more physiological 3D, matrix-based cell culture techniques were applied for relevant endpoints exploration such as clonogenic survival and DNA damage. The functional characteristics of the total pool of examined targets involved interactions with integrins, the cytoskeleton, cancer related signal transduction and DNA repair (Fig. 1B, left). In Fig. 1C, we show the significant cytotoxic and radiosensitizing effects as well as enhanced DNA damage of the top 30 targeted genes in 35 cell models across six cancer types most widely used in the Cordes lab. A complete collection of experimental results is given in Fig. S1 and Table S1. The most prominent adhesion related cancer targets, whose inhibition caused cytotoxicity and radiosensitization, were integrin β1 (ITGB1), Focal adhesion kinase (FAK; PTK2) and Particular Interesting New Cysteine-Histidine rich protein (PINCH1; LIMS1). Additionally, the Epidermal growth factor receptor (EGFR) and several DNA damage response related proteins like DNA-PKcs (PRKDC), Poly(ADP-Ribose) Polymerase 1 (PARP1) and Ataxia Telangiectasia Mutated (ATM) are among the top targets. In order to validate the observed Cordes lab perturbation effects, an extensive PubMed literature search on the top 30 targets (see Fig. 1C), comprising a total of 1531 publications, was conducted. We observed that many adhesion associated genes, like LIMS1 (PINCH), ILK, or specific integrins, have only been marginally studied as cancer targets in radiation research (Fig. S2, Table S1). For one of our prime targets, ITGB1, we obtained confirmatory cytotoxic and radiosensitizing effects upon deactivation in a total of 15 studies. Similar validations were reported for other genes like PTK2 (Focal adhesion kinase), HDAC1, EGFR, PARP1/2 or ATM. Taken together, this literature search showed many adhesion associated targets to be underexplored but with a high potential for therapeutic exploitability. Overall, this accumulated knowledge prompted us to hypothesize that combining our in vitro results with large publicly available cancer patient databases and network-based analyses uncovers new promising targets for radiotherapeutic-relevant adhesome targeting.

### 3.3 Nine cancers with highly heterogenous transcriptomes share similar expression changes of adhesion associated genes compared to the corresponding normal tissues

**Fig. 2:**
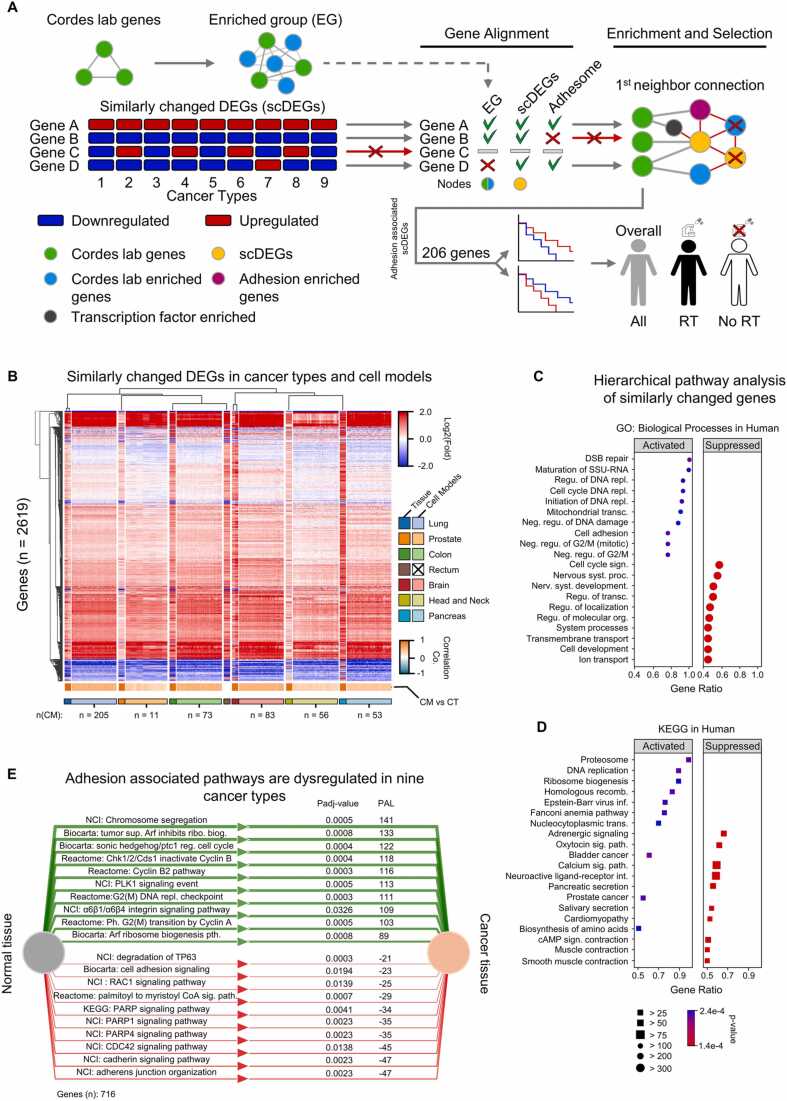
Nine cancer types with highly heterogenous transcriptomes share similar expression changes of adhesion associated genes compared to the corresponding normal tissues. (A) Schematic of the study workflow with all gene comparisons and selection processes. (B) Similarly changed DEGs (scDEGs) in different cancer types (CTs) and cell models (CMs, total number of included CMs indicated below the heatmap). CMs were aligned with hierarchically clustered 9 CTs. Correlation analysis compared scDEGs of CTs and CMs. (C-D) Highly enriched GO biological processes (C) and KEGG pathways (D) shared among cancer types and ordered according to gene enrichment scores. The color coding indicates the significance level. The symbol size represents the gene number. (E) Adhesion associated scDEGs pathway activation chart (OncoBoxPD). Green lines show the top 10 activated pathways; red lines show the top 10 inhibited pathways ordered based on absolute value of pathway activation levels (PALs). PAL values and FDR-adjusted p-values are shown right to the pathway names. LUSC, Lung squamous cell carcinoma; LUAD, Lung adenocarcinoma; PRAD, Prostate adenocarcinoma; COAD, Colon adenocarcinoma; READ, Rectum adenocarcinoma; LGG, Lower grade glioma; GBM, Glioblastoma multiforme; HNSCC, Head and neck squamous cell carcinoma; PAAD, Pancreatic adenocarcinoma.

We comparatively investigated differently expressed genes (DEGs) in 9 cancer types and their corresponding normal tissues and detected 27253 significantly overlapping coding and non-coding genes between squamous cell carcinoma of the lung (LUSC), LUAD, PRAD, COAD, adenocarcinoma of the rectum (READ), brain lower grade glioma (LGG), GBM, HNSCC and PAAD (Fig. S3A). Using principal component analysis of all overlapping genes (fold changes), we clearly identified variations between the 9 cancers with PAAD being the most variable (Fig. S3B). Therefore, only DEGs demonstrating similar expression changes found in at least 8 out of the 9 cancer types were considered for further analysis, hereafter named similarly changed DEGs (scDEGs, Fig. S3C). Of note, out of the 2619 genes fitting the criteria, 89% were upregulated and 11% were downregulated (Table S2). Additionally, we acquired the DEG profiles of the corresponding cancer cell models and aligned them to the scDEGs with further hierarchical clustering (Fig. 2B). Intriguingly, the heatmap pinpoints towards the similarity between cancer tissue DEGs and cancer cell model DEGs fold changes, which is shown by more than 50% positive correlations (Fig. 2B, Table S2). The next step involved an overlapping of the enriched genes acquired from Cordes lab publications with the scDEGs dataset from Fig. 2B. More specifically, we identified adhesion associated scDEGs (workflow in Fig. 2 A), which overlapped with the Cordes lab genes (i.e. 212) and enriched them with the most closely associated transcription factors and adhesion genes (Table S2). We further selected genes with a minimum of 2 degrees (connections) in the network and DEGs with an average fold change of either> 1.5 or< 0.5 from normal tissue for subsequent survival analyses. A total of 206 genes were passing these criteria and formed our initial pool of adhesion associated scDEGs (Table S2). Taken together, we demonstrated similarities in DEGs between cancer cell models and biopsies taken from patients of 9 genetically diverse cancer types as well as a great overlap in adhesion associated gene profiles indicative of a fundamental involvement of these genes in the chosen cancer types.

### 3.4 Highly enriched biological processes and pathways are shared among different cancer types

The scDEGs found in the 9 cancers (Fig. 2B) were implemented in GO and KEGG pathway analyses. Hierarchical clustering revealed significant and highly enriched biological processes and pathways connected to cancer development and progression (Fig. 2C-D). This revealed that the 9 cancer types utilize overactivated cell adhesion, DNA repair, and cell cycle-dependent DNA replication on the one hand, and suppress hormonal, cell cycle signaling, localization, and general systemic processes on the other. To investigate the biological properties of DEGs without similar alterations in different cancer types (non-scDEGs), we performed an overrepresentation analysis using the KEGG, GO, and Reactome databases (Fig. S4). Our results showed that non-scDEGs exerted cancer-type-specific functions. For example, genes involved in determining the extracellular matrix were highly upregulated in HNSCC compared with the corresponding normal tissue and other cancer types. Similarly, lung and brain tumors exhibited different immune-related signatures relative with other cancers. Tissue-specific downregulated gene sets were also observed, such as decreased neuronal genes in brain tumors or decreased pancreatic secretion in PAAD. Overall, these enrichment analyses revealed similar functional changes between the nine cancer types studied and the corresponding normal tissues. Furthermore, we indicated the characteristic traits which differentiate the cancer types and enable functional subgrouping.

### 3.5 Adhesion associated pathways are dysregulated in nine cancer types

To deeper understand the shared processes and pathways of our selected adhesion associated scDEGs, we first reconstructed the 1st neighbor network between these 206 genes and total scDEGs (Table S2), which resulted ultimately in primary and secondary connections with 64% of scDEGs. OncoBoxPD functional enrichment analysis of the genes in this network allowed us to equally assess multiple pathway databases (Fig. 2E, Table S4). This demonstrated that α6β1/α6β4 integrin signaling together with Arf associated pathways were found to be activated, whereas regulators of diverse cellular responses, including cell cycling, survival, and differentiation were suppressed in the 9 cancer types relative to their corresponding normal tissues.

### 3.6 Combined adhesion associated scDEGs and TCGA patient survival analysis identifies genes of interest

**Fig. 3:**
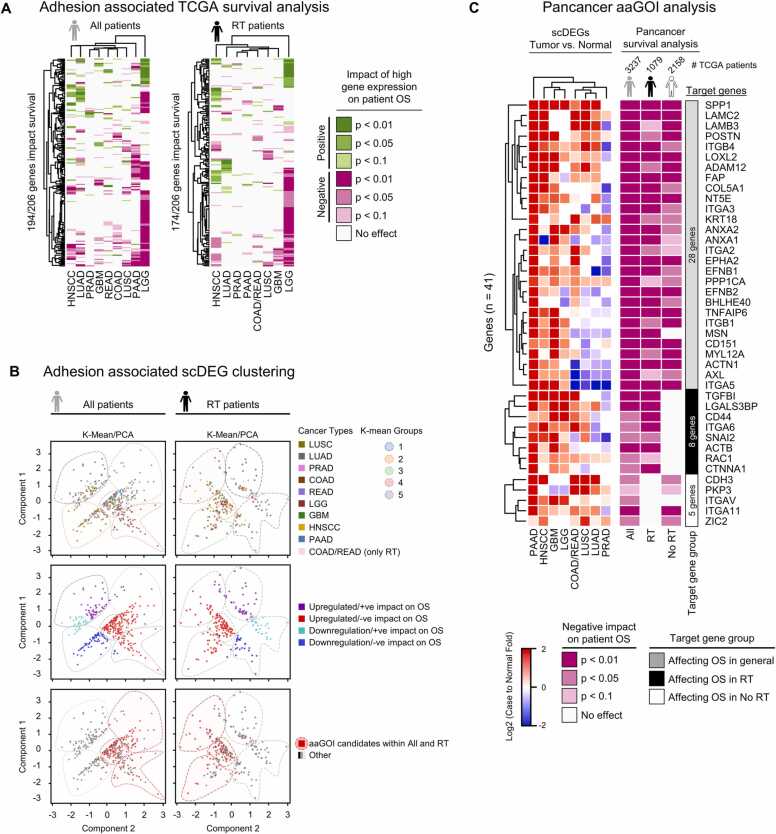
Combined adhesion associated scDEGs and TCGA patient survival analyses identify genes of interest. (A) TCGA patient survival analysis of adhesion associated scDEGs (206 genes) in 9 cancer types. For each gene, patients of the respective TCGA studies were divided in high and low expression cohorts (group cutoff: median expression) and subjected to Kaplan-Maier analysis. The heatmaps highlight the impact of the significantly overexpressed adhesion associated genes on overall survival (OS) of patients by log-rank test significance cutoffs (p < 0.1; p < 0.05; p < 0.01). The left heatmap includes all patients (“All”) of the respective cancer-type cohorts. The right heatmap selects only for radiotherapy (“RT”)-treated patients. Genes with no effect across all cancer types were excluded. Cancer types COAD and READ were combined for the “RT” cohort analysis due to low patient numbers. (B) Combined K-mean / PCA data analysis of adhesion associated scDEGs and corresponding reciprocal p-values for TCGA patient survival. Data sets were grouped into positive and negative categories according to their impact on “All” (left column) and “RT” (right column) survival. Individual points of top row graphs indicate the respective cancer type. Localization of upregulated and downregulated genes with respective positive or negative impacts on survival (middle). Selected genes of interest (bottom; aaGOI within “All” and “RT”) include only overexpressed genes with a negative impact on OS in at least two cancer types. (C) Pancancer target gene analysis of aaGOIs (n = 41) impacting OS in 9 TCGA cancer cohorts. The OS in the “RT”- and “No RT”-subcohorts divides the GOIs into three groups, primarily affecting OS in either “All” (grey, 28 genes), “RT” (8 genes, black), or “No RT” (5 genes, white) cohorts. Each group was independently hierarchically clustered based on its expression change to normal tissue across the respective cancer types. aaGOI, adhesion associated gene of interest. LUSC, Lung squamous cell carcinoma; LUAD, Lung adenocarcinoma; PRAD, Prostate adenocarcinoma; COAD, Colon adenocarcinoma; READ, Rectum adenocarcinoma; LGG, Lower grade glioma; GBM, Glioblastoma multiforme; HNSCC, Head and neck squamous cell carcinoma; PAAD, Pancreatic adenocarcinoma.

Next, we took the aforementioned identified 206 genes, termed adhesion associated scDEGs, to evaluate them in the 9 selected cancer types for their clinical significance by a TCGA patient survival analysis (Fig. 3A). The individual impact of high gene expression on patient overall survival (OS) was assessed by combining the Kaplan-Maier analysis with the specific OS impact directions (positive: prolonged OS; negative: shortened OS). Analyses were performed for all TCGA patient cohorts of the investigated cancer types (“All”) and the corresponding subcohort radio(chemo)therapy only (“RT”) to discover radiotherapy-specific survival responses. In the “All” survival analyses (Fig. 3A, left heatmap), 194 out of the 206 target genes affected patient survival in the frame of our log-rank test significance cutoffs (p < 0.1; p < 0.05; p < 0.01). The greatest reduction in OS was observed in patients suffering from LGG in clear contrast to, for example, HNSCC patients. Hierarchical clustering pointed out specific subgroups of genes, which impacted OS across the majority of cancer types. The top 5 identified genes affecting survival negatively (p < 0.05) in at least 4/9 cancer types were LOXL2, ITGA3, ACTB, EFNB2 and ITGB1. In contrast, our analyses also revealed genes prolonging patient OS at high expression such as RXRB3, SMAD9, SATB1, CD24 and TBP. Regarding the “RT” patient subcohort (Fig. 3A, right heatmap), a total of 174 genes were affecting “RT” survival. High expression of CD151, EFNB2, COL5A1, ITGA3 or ANAX2 most significantly reduced OS, while high SOX4, CX3CR1, MYB, TCF7L2 and XBP1 expression levels correlated with prolonged “RT” OS in multiple cancer types (TCGA survival analysis source data provided in Table S3).

To aid target gene selection of common and radiotherapy related regulators, we conducted integrated K-Mean / PCA analysis on TCGA patient OS from Fig. 3A with their corresponding fold change expression of the respective cancer types to normal tissue. These analyses showed predominantly characteristic data clustering for both groups (Fig. 3B, Fig. S5A-B). Furthermore, most cancer types were found to be equally distributed between each identified cluster (Fig. 3B, top). The data relationship between the fold change expression of adhesion associated scDEGs and patient OS were mapped back to the K-Mean clusters to identify the clusters including potential genes of interest (GOI; Fig. 3B, middle). Clusters 3–5 in group “All” and 2–4 in “RT” were exclusively allocating the data with upregulated gene changes and negative impact on patients` OS for both subgroups. Genes found within these clusters are potential candidates for molecular targeted interventions.

**Fig. 4:**
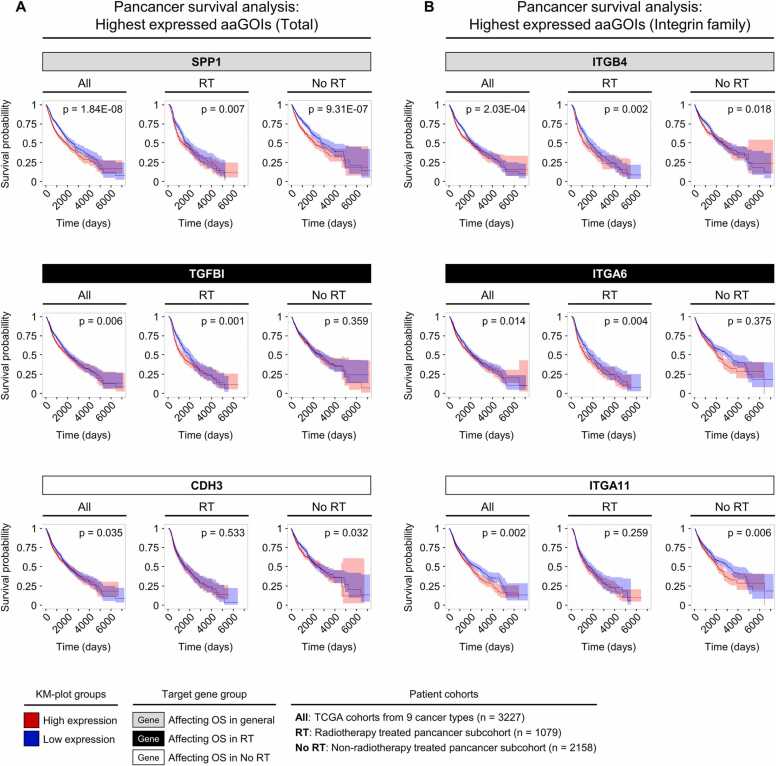
Pancancer survival analyses of highly overexpressed adhesion associated genes of interest. Candidate genes derived from each target gene group of Fig. 3 C were included in individual pancancer overall survival analyses of either “All” patients (n = 3227), “RT” (n = 1079) or “No RT” (n = 2158) subcohorts after initial diagnosis. Survival plots in (A) represent adhesion associated genes of interest (aaGOIs) with highest overall expression fold change relative to normal tissue of each group. (B) Top aaGOIs of the integrin family across the defined target gene groups. Median expression defined the cutoff between high (red) and low (blue) expression survival curves. (A-B) Log-rank test p-values and confidence intervals are indicated.

We derived our adhesion associated genes of interest (aaGOIs) from overlapping “All” and “RT” clustering analyses (Fig. 3B, bottom) with the following criteria: (i) we included all genes which showed an overexpression compared to normal tissue in a particular cancer type paired with low patients` OS (i.e. negative impact); (ii) we excluded all of the selected genes which mediated prolonged patient OS (p < 0.05) in one or more cancer types. Subsequently, all patients of the 9 TCGA cancer type cohorts analyzed in the present study (n = 3237) were combined in a pancancer survival analysis; (iii) for the final, third filtering step, we kept only genes which showed a significant pancancer patients` OS shortening (i.e. negative impact; p < 0.1). In total, we identified 41 aaGOIs (Fig. 3C). To unravel the potential clinical impact of our aaGOIs for radiotherapy, the pancancer subcohorts “RT” (n = 1079) and “No RT” (n = 2158) were additionally examined for patients` OS shortening under high aaGOI expression. This subcohort segmentation allowed us to group the aaGOIs into three categories: (i) 28 genes affected the OS in all patients independent of therapy type; (ii) 8 aaGOIs genes primarily influenced “RT” patients; (iii) 5 genes mainly affected the “No RT” cohort (Fig. 3C). The impact of each aaGOI on patients` OS for each cancer type is shown in Fig. S6. Each of the three groups were hierarchically clustered based on the average fold change to normal tissue, highlighting the consistently overexpressed aaGOIs across all examined cancer types. The individual pancancer Kaplan-Maier survival analyses for the highest expressed aaGOIs are visualized in Fig. 4. The top candidates from each target gene group were Secreted Phosphoprotein 1 (SPP1, “All” group), Transforming Growth Factor Beta Induced (TGFBI, “RT” group) and Cadherin 3 (CDH3, “No RT” group) (Fig. 4 A). The top integrins were ITGB4 (“All” group), ITGA6 (“RT” group) and ITGA11 (“No RT” group) (Fig. 4B). Taken together, these results pinpoint the importance of adhesion associated genes across a wide range of highly heterogenous cancer types. The identified aaGOIs demonstrated similar upregulation and impact on patients` OS.

### 3.7 Integrin signaling pathways negatively affect the efficacy of conventional radio(chemo)therapy in the studied cancer types

**Fig. 5:**
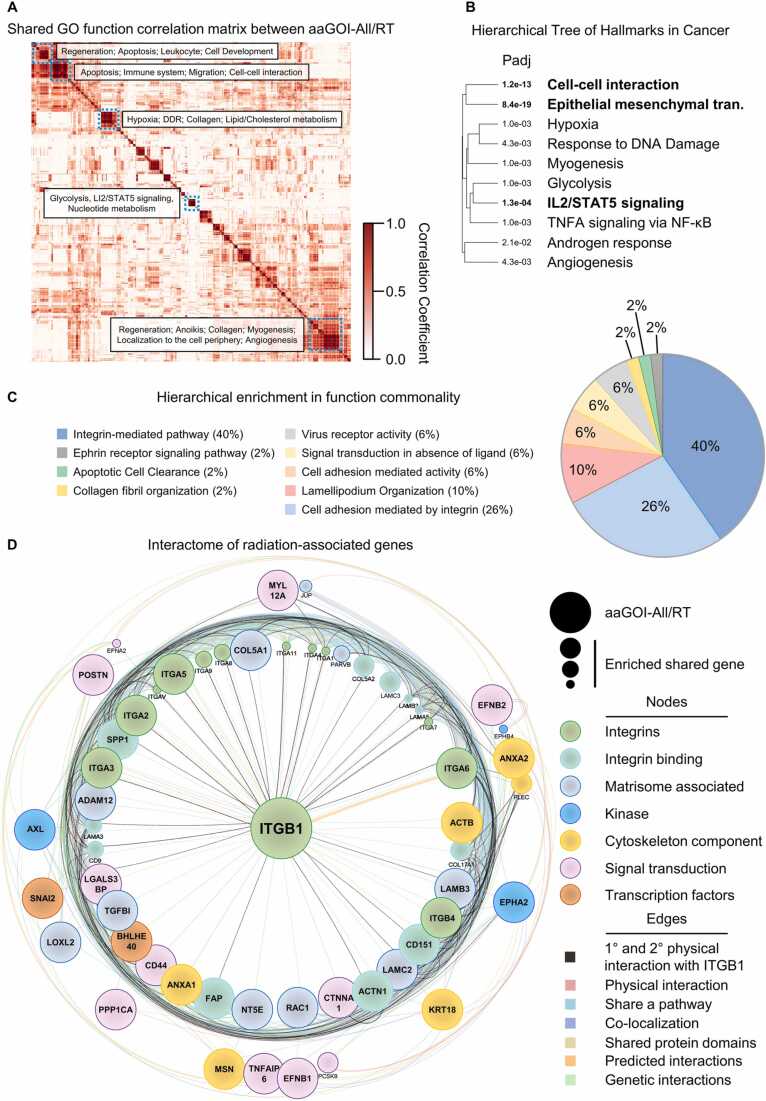
Overexpressed genes associated with integrin pathways negatively affect patient survival across 9 cancer types. (A) Pairwise clustering correlation evaluation of significant (p < 0.05; FDR<0.25) functional associations (1614 terms, acquired from Wikipathways, Reactome, KEGG, Gene Ontology (GO), Molecular Signatures Database (MSigDB) resources; proceeded with g:profiler, ShinyGO, GSEA) with “All” and “RT” target gene groups from Fig. 3C (aaGOI-All/RT, n = 36). The blue broken line indicates the largest clustered terms with a correlation coefficient of>0.75. (B) Hierarchical clustering tree of the MSigDB hallmarks in cancer acquired from (A). (C) Hierarchical clustering of the assigned GO terms referring to functional commonality based on ClueGO network analysis for aaGOI-All/RT (D) Constructed interaction network via GeneMANIA with the closest interrelated genes for the aaGOI-All/RT groups. The color code of the nodes indicates the GO of molecular functions (processed with ShinyGO). The size of the nodes is proportional to the gene score assessed by GeneMANIA. The lines connect nodes (edges) with considered relationship. Radial structures indicate an increase of the interconnection enrichment from center to edge. aaGOI, adhesion associated genes of interest.

Next, we investigated the shared treatment-perturbing and OS-reducing impact potential of the selected 36 aaGOIs from the groups “All” and “RT” (aaGOIs-All/RT) in the 9 different cancer types. To acquire knowledge about the underlying processes in the identified aaGOIs-All/RT group, we performed a pathway enrichment analysis. Positive pairwise clustering correlation comparisons of significantly (p < 0.05; FDR<0.25) associated 1614 functional terms acquired from multiple databases revealed that aaGOIs-All/RT are involved in key cellular processes such as cell death mechanisms, regeneration, immune system and localization regulations (Fig. 5A, Table S4). Further hierarchical clustering of MSigDB hallmarks identified in the correlation matrix uncovered highly significant associations with the development of peripheral immune cells, transformation and enhanced migration characteristics accompanied by epithelial mesenchymal transition, cell-cell interaction and IL2/STAT5 signaling in cancers (Fig. 5B). Intriguingly, independent functional network pathway analysis (ClueGO) based on shared functional enrichment commonalities revealed that 66% of aaGOIs-All/RT functioning in integrin related pathways and additional 34% are associated to cell adhesion, fiber organization and apoptotic processes (Fig. 5C). After reconstructing biological interactome networks based on the overexpression hierarchical gene order, we observed ITGB1 as the central node physically connecting 60% of genes with primary and secondary protein-protein interactions (Fig. 5D). Overall, our findings highlight: (i) a strong association between our 36 aaGOIs-All/RT and general key cancer cell functions, and (ii) β1 integrin related pathways are predicted to be central to these associations.

## 4 Discussion

Intra- and intertumoral heterogeneity are extensive with multi-layered disparities affecting numerous fundamental processes like survival, proliferation, and invasion [2]. Consequently, different cancers demonstrate varying efficacy to both conventional and novel molecular targeted approaches [4]. Therapeutic improvements with conventional radio(chemo)therapy have therefore plateaued across different cancer types in the last decades [44], [45]. In addition to a myriad of intra- and extracellular factors of resistance [5], extensive work of the Cordes lab and others showed the fundamental role of the adhesome in cancer [7], [14], [46]. Here, we evaluated the reasonability and relevance of the preclinical Cordes lab datasets in a pancancer context using large publicly available transcriptomic and patient survival databases. We found that a large set of genes is similarly changed across highly heterogenous cancer types compared to the corresponding normal tissue. Connecting these similarly changed DEGs (scDEGs) with the Cordes lab adhesion associated targets revealed groups of genes whose overexpression impacted generally or radio(chemo)therapy-specifically patient survival. Functional analyses classified these genes as being involved in key cancer pathways, which are connected through integrin related signaling.

In the past decades, multiple large patient cohort studies and advances in next-generation omics analytics revealed diverse prosurvival mechanisms within individual cancer types and cancer cell models [23], [26], [47]. These massive databases hold a great potential for investigating shared and conserved biochemical adhesion related cues. These cues are profoundly modified in cancer cells, in which cell-ECM interactions are not only altered by adverse mechanobiology but also participate in mediating therapy resistance in cancer. In this study, we focused on non-small cell lung cancer (LUAD, LUSC) [20], [48], colorectal cancer (COAD, READ) [49], [50], brain cancers (GBM, LGG) [18], [51], head and neck cancer (HNSCC) [52], [53], pancreatic cancer (PAAD) [54], [55], and prostate cancer (PRAD) [16], in which we and others reported therapy sensitization by targeting focal adhesion proteins.

To connect these promising preclinical observations with cancer patient transcriptomics, we took all adhesion related targets explored in the Cordes lab as starting point of our analysis. The bias of this one-lab gene set selection was addressed in a literature search on the 30 most thoroughly investigated targets, which confirmed radiosensitizing effects in terms of cell survival and DNA damage response. Combined bioinformatic analyses across cancer types and their corresponding cell models allowed us to identify shared DEGs within at least eight cancer types. We found 2619 scDEGs associated with activation of DNA repair, DNA replication, and adhesion as well as suppression of cell cycle signaling, localization, and general systemic processes. Interestingly, with around 60% correlation, DEGs found in these tumor tissues are shared with the corresponding cancer cell models. Previous bioinformatic approaches similar to the presented one were mainly conducted on either individual gene sets or in specific cancer types [56], [57]. Furthermore, in these studies the impact on patient survival or the intermolecular interactions were often not taken into consideration. For the first time, as to our knowledge, similarly changed DEGs were described between the examined 9 cancer types and analyzed in the context of the cancer adhesome.

To focus on adhesion genes, we interconnected the Cordes lab gene sets with identified scDEGs, which resulted in 206 adhesion associated scDEGs. Their clinical relevance was assessed by comprehensive TCGA survival and tumor overexpression analyses. We ultimately found 36 genes of interest (aaGOIs-All/RT), which impacted survival of either all patients across multiple cancer types or exclusively in the radio(chemo)therapy treated cohort. These genes were functionally involved in cell death mechanisms, regeneration, the immune system, and enhanced migration characteristics accompanied by epithelial-mesenchymal transition (EMT) processes. In line, comparable and commonly dysregulated cancer functionalities, like apoptosis or actin-cytoskeleton regulation, were also discovered in a recent pancancer multi-omics study [58]. Network analysis of the identified aaGOIs-All/RT revealed a large dependency on the integrin signaling axis. In fact, of the 36 genes, 60% were either integrins or their 1st neighbor's physical interactors. Integrin β1 emerged as the central node with a maximum of gene interconnections to α integrins and numerous other focal adhesion and ECM related proteins. These findings validate the reasonability of the Cordes lab work on the cancer adhesion resistome. In fact, integrin β1 was the most investigated cancer target in the group over the years [15], [48], [59], [60], [61]. The importance of this integrin is emphasized by a large body of preclinical studies on its therapy sensitizing potential [17], [20], [62]. By linking preclinical results with pancancer datasets, integrin β1 and its interacting proteins revealed a negative impact on patient survival, clearly pinpointing the significance of this signaling hub for inherent and/or acquired resistance as well as precision medicine. In line with this, an integrin β1 targeting antibody for the treatment of high-grade glioma patients just entered clinical trials accompanied by real time imaging to assess its pharmacokinetics (NCT04608812). This and other novel optimized integrin related therapeutics reentered clinical trials after the failure of αV-integrin targeting peptidomimetics in GBM [63].

We identified β4 and α6 integrin as the most overexpressed integrins in our study, adversely affecting survival of either all (β4) or primarily radio(chemo)therapy treated patients (α6). Previous in vitro experiments of our lab reflect this pattern, where general cytotoxic and radiosensitizing effects were observed upon integrin β4 [54] and integrin α6 inhibition [52], [64]. Research on glioblastoma and breast cancer supports the suggested pancancer involvement of integrin α6 in radioresistance [65], [66]. Concerning integrin β4, a connection to EMT could be observed [67], which was also identified as a hallmark ontology in our final aaGOI-All/RT set. Likewise, both of these integrins and their heterodimeric α6β4 receptors were found to be intricately involved in cancer stem cell (CSC) properties [46] and their deactivation demonstrated effective perturbation of CSC populations [68], [69].

Beside integrins, our meta-analysis revealed promising adhesion associated genes highly deregulated across multiple cancer types. Especially the pancancer overexpression of Osteopontin (SPP1) and its impact on survival was remarkable and increasing evidence delineates SPP1 as a critical factor in tumorigenesis, metastasis, immune response and chemoresistance [70]. Importantly, SPP1 is a direct integrin interactor, which underpins the involvement of this signaling axis in adverse pancancer adhesome signaling and potential targeting opportunities. Regarding our aaGOIs primarily impacting the survival of radiotherapy treated patients, transforming growth factor-beta-induced (TGFBI) stood out in particular. Its primary role consists in connecting various integrins to the ECM. However, a growing body of research points towards its two-edged role in regulating tumor progression and resistance [71], [72]. Central to radio(chemo)therapy was the discovery that TGFBI mediates a survival advantage by modifying DNA damage repair [73]. Furthermore, it counteracts the radio(chemo)sensitizing potential of targeting the adhesion associated receptor DDR1 [74], [75].

Intriguingly, at least half of our aaGOIs-All/RT were identified in tumor extracellular vesicles (EVs) from primary or metastatic sites in colorectal cancer [76]. This and other studies provide preliminary evidence of the utilization of EV associated integrins as potential biomarkers for detecting cancer, tumor stage or metastasis, where particularly integrin β1 and α6 were found to be of high importance [77]. However, such EVs contribute also to therapy resistance [78] and were presumably a critical factor why previous clinical trials addressing integrin targeting displayed low efficacy [63]. This underscores the need for the clarification of three aspects: (i) which of the various adhesome targets (like integrins, SPP1, TGFBI, AXL, EPHA2, LOXL, ect.) are key determinants in a specific cancer type; (ii) what is the therapy sensitizing potential of targeting these key determinants; (iii) which multi-targeting approaches are required in a specific cancer type to hamper the numerous bypass opportunities leading to low treatment efficacy [7].

Overall, the multiple filtering steps in our adhesion associated meta-analysis retained key integrins and interaction partners suggesting a conserved role across heterogeneous cancer types. This implies that interconnecting the identified hallmark in cancer characteristics with adhesion associated inhibition approaches could reveal previously undetected preserved prosurvival mechanisms driving normal tissues into cancer. Connecting adhesome and matrisome [79], [80], the application of in-silico methodologies on the individual protein level [81] combined with multi-omics pancancer analyses will provide further proofs needed in bringing promising adhesion associated anti-cancer therapies from bench to bedside.

## 5 Conclusion

In conclusion, our meta-analysis shows a clear involvement of integrins, especially β1, β4 and α6, in adverse cancer therapy outcomes indicating a conserved role of integrin adhesome signaling across the heterogenous cancer types. A set of our identified genes (aaGOI-RT) specifically impacted the survival of radio(chemo)therapy-treated patients, offering potential new approaches for research in molecular-targeted strategies. The relevance and reasonability of the Cordes lab investigations on the cancer adhesion resistome are confirmed by our meta-analysis since key preclinical candidates displayed aberrant expression patterns and adverse impacts on patient survival in a pancancer context. Future in-silico and experimental research is warranted to uncover the role of our identified gene candidates in the cancer adhesome and their therapeutic potential for molecular interventions. The involvement of highly expressed integrins and their interconnectors in the cancer resistome could contribute to pave the way towards personalization and precision oncology.

## Funding statement

This work was supported by the 10.13039/501100005972German Cancer Aid (70113293 to N.C.), the European Union‘s Horizon 2020 research and innovation program under Marie Skłodowska-Curie grant agreement (860245 – Theradnet to N.C.), and the Saxon Ministry of Science and Arts and the EFRE Europäische Fonds für regionale Entwicklung, Europa fördert Sachsen (100066308 to N.C.).

## CRediT authorship contribution statement

**Olegs Borodins**: Investigation, Software, Formal analysis, Conceptualization, Writing − original draft, Writing − review & editing. **Felix Broghammer**: Investigation, Software, Formal analysis, Conceptualization, Writing − original draft, Writing − review & editing. **Michael Seifert**: Supervision, Writing − review & editing. **Nils Cordes**: Conceptualization, Supervision, Funding acquisition, Writing − original draft, Writing − review & editing.

## Conflict of interest

The authors declare no competing interests.

## References

[1] Ferlay J., Colombet Murielle, Soerjomataram I., Donald Parkin M., Piñeros M. (2021). Cancer statistics for the year 2020: An overview. Int J Cancer.

[2] Marusyk A., Almendro V., Polyak K. (2012). Intra-tumour heterogeneity: a looking glass for cancer. Nat Rev Cancer.

[3] Jamal-Hanjani M., Quezada S.A., Larkin J., Swanton C. (2015). Translational implications of tumor heterogeneity. Clin Cancer Res.

[4] Marusyk A., Janiszewska M., Polyak K. (2020). Intratumor heterogeneity: the rosetta stone of therapy resistance. Cancer Cell.

[5] Vasan N., Baselga J., Hyman D.M. (2019). A view on drug resistance in cancer. Nat 2019 5757782.

[6] Jurj A., Ionescu C., Berindan-Neagoe I., Braicu C. (2022). The extracellular matrix alteration, implication in modulation of drug resistance mechanism: friends or foes?. J Exp Clin Cancer Res.

[7] Dickreuter E., Cordes N. (2017). The cancer cell adhesion resistome: Mechanisms, targeting and translational approaches. Biol Chem.

[8] Winograd-Katz S.E., Fässler R., Geiger B., Legate K.R. (2014). The integrin adhesome: from genes and proteins to human disease. Nat Rev Mol Cell Biol.

[9] Horton E.R., Byron A., Askari J.A., Ng D.H.J., Millon-Frémillon A., Robertson J. (2015). Definition of a consensus integrin adhesome and its dynamics during adhesion complex assembly and disassembly. Nat Cell Biol.

[10] Wozniak M.A., Modzelewska K., Kwong L., Keely P.J. (2004). Focal adhesion regulation of cell behavior. Biochim Biophys Acta - Mol Cell Res.

[11] Humphries J.D., Chastney M.R., Askari J.A., Humphries M.J. (2019). Signal transduction via integrin adhesion complexes. Curr Opin Cell Biol.

[12] Geiger B., Spatz J.P., Bershadsky A.D. (2009). Environmental sensing through focal adhesions. Nat Rev Mol Cell Biol.

[13] Eke I., Cordes N. (2015). Focal adhesion signaling and therapy resistance in cancer. Semin Cancer Biol.

[14] Pickup M.W., Mouw J.K., Weaver V.M. (2014). The extracellular matrix modulates the hallmarks of cancer. EMBO Rep.

[15] Eke I., Zscheppang K., Dickreuter E., Hickmann L., Mazzeo E., Unger K. (2015). Simultaneous β1 integrin-EGFR targeting and radiosensitization of human head and neck cancer. J Natl Cancer Inst.

[16] Lee Y.C., Jin J.K., Cheng C.J., Huang C.F., Song J.H., Huang M. (2013). Targeting constitutively activated β1 integrins inhibits prostate cancer metastasis. Mol Cancer Res.

[17] Hanker A.B., Estrada M.V., Bianchini G., Moore P.D., Zhao J., Cheng F. (2017). Extracellular Matrix/Integrin Signaling Promotes Resistance to Combined Inhibition of HER2 and PI3K in HER2+ Breast Cancer. Cancer Res.

[18] Korovina I., Vehlow A., Temme A., Cordes N. (2022). Targeting integrin α2 as potential strategy for radiochemosensitization of glioblastoma. Neuro Oncol.

[19] Zienert E., Eke I., Aust D., Cordes N. (2015). LIM-only protein FHL2 critically determines survival and radioresistance of pancreatic cancer cells. Cancer Lett.

[20] Kanda R., Kawahara A., Watari K., Murakami Y., Sonoda K., Maeda M. (2013). Erlotinib resistance in lung cancer cells mediated by integrin β1/Src/Akt-driven bypass signaling. Cancer Res.

[21] Rossow L., Eke I., Dickreuter E., Cordes N. (2015). Targeting of the EGFR/β1 integrin connecting proteins PINCH1 and Nck2 radiosensitizes three-dimensional SCC cell cultures. Oncol Rep.

[22] de la Puente P., Weisberg E., Muz B., Nonami A., Luderer M., Stone R.M. (2015). Identification of ILK as a novel therapeutic target for acute and chronic myeloid leukemia. Leuk Res.

[23] Ma X., Liu Y., Liu Y., Alexandrov L.B., Edmonson M.N., Gawad C. (2018). Pan-cancer genome and transcriptome analyses of 1,699 paediatric leukaemias and solid tumours. Nat 2018.

[24] Weinstein J.N., Collisson E.A., Mills G.B., Shaw K.R.M., Ozenberger B.A., Ellrott K. (2013). The cancer genome Atlas pan-cancer analysis project. Nat Genet.

[25] Tang Z., Li C., Kang B., Gao G., Li C., Zhang Z. (2017). GEPIA: a web server for cancer and normal gene expression profiling and interactive analyses. Nucleic Acids Res.

[26] Ghandi M., Huang F.W., Jané-Valbuena J., Kryukov G.V., Lo C.C., McDonald E.R. (2019). Next-generation characterization of the Cancer Cell Line Encyclopedia. Nat 2019 5697757.

[27] Zolotovskaia M.A., Tkachev V.S., Guryanova A.A., Simonov A.M., Raevskiy M.M., Efimov V.V. (2022). OncoboxPD: human 51 672 molecular pathways database with tools for activity calculating and visualization. Comput Struct Biotechnol J.

[28] Raudvere U., Kolberg L., Kuzmin I., Arak T., Adler P., Peterson H. (2019). g:Profiler: a web server for functional enrichment analysis and conversions of gene lists (2019 update). Nucleic Acids Res.

[29] Lambert S.A., Jolma A., Campitelli L.F., Das P.K., Yin Y., Albu M. (2018). The human transcription factors. Cell.

[30] Shannon P., Markiel A., Ozier O., Baliga N.S., Wang J.T., Ramage D. (2003). Cytoscape: a software environment for integrated models of biomolecular interaction networks. Genome Res.

[31] Warde-Farley D., Donaldson S.L., Comes O., Zuberi K., Badrawi R., Chao P. (2010). The GeneMANIA prediction server: biological network integration for gene prioritization and predicting gene function. Nucleic Acids Res.

[32] Szklarczyk D., Gable A.L., Lyon D., Junge A., Wyder S., Huerta-Cepas J. (2019). STRING v11: protein-protein association networks with increased coverage, supporting functional discovery in genome-wide experimental datasets. Nucleic Acids Res.

[33] Carbon S., Douglass E., Good B.M., Unni D.R., Harris N.L., Mungall C.J. (2021). The Gene Ontology resource: enriching a GOld mine. Nucleic Acids Res.

[34] Goldman M.J., Craft B., Hastie M., Repečka K., McDade F., Kamath A. (2020). Visualizing and interpreting cancer genomics data via the Xena platform. Nat Biotechnol.

[35] Kanehisa M., Furumichi M., Tanabe M., Sato Y., Morishima K. (2017). KEGG: new perspectives on genomes, pathways, diseases and drugs. Nucleic Acids Res.

[36] Martens M., Ammar A., Riutta A., Waagmeester A., Slenter D.N., Hanspers K. (2021). WikiPathways: connecting communities. Nucleic Acids Res.

[37] Gillespie M., Jassal B., Stephan R., Milacic M., Rothfels K., Senff-Ribeiro A. (2022). The reactome pathway knowledgebase 2022. Nucleic Acids Res.

[38] Subramanian A., Tamayo P., Mootha V.K., Mukherjee S., Ebert B.L., Gillette M.A. (2005). Gene set enrichment analysis: a knowledge-based approach for interpreting genome-wide expression profiles. Proc Natl Acad Sci USA.

[39] Ge S.X., Jung D., Jung D., Yao R. (2020). ShinyGO: a graphical gene-set enrichment tool for animals and plants. Bioinformatics.

[40] Liberzon A., Birger C., Thorvaldsdóttir H., Ghandi M., Mesirov J.P., Tamayo P. (2015). The Molecular Signatures Database (MSigDB) hallmark gene set collection. Cell Syst.

[41] Bindea G., Mlecnik B., Hackl H., Charoentong P., Tosolini M., Kirilovsky A. (2009). ClueGO: a Cytoscape plug-in to decipher functionally grouped gene ontology and pathway annotation networks. Bioinformatics.

[42] Hunter J.D. (2007). Matplotlib: A 2D graphics environment. Comput Sci Eng.

[43] Virtanen P., Gommers R., Oliphant T.E., Haberland M., Reddy T., Cournapeau D. (2020). SciPy 1.0: fundamental algorithms for scientific computing in Python. Nat Methods.

[44] Bagnyukova T., Serebriiskii I.G., Zhou Y., Hopper-Borge E.A., Golemis E.A., Astsaturov I. (2010). Chemotherapy and signaling: How can targeted therapies supercharge cytotoxic agents. Cancer Biol Ther.

[45] Walters S., Maringe C., Coleman M.P., Peake M.D., Butler J., Young N. (2013). Lung cancer survival and stage at diagnosis in Australia, Canada, Denmark, Norway, Sweden and the UK: a population-based study, 2004-2007. Thorax.

[46] Cooper J., Giancotti F.G. (2019). Integrin signaling in cancer: mechanotransduction, stemness, epithelial plasticity, and therapeutic resistance. Cancer Cell.

[47] Sanchez-Vega F., Mina M., Armenia J., Chatila W.K., Luna A., La K.C. (2018). Oncogenic signaling pathways in the cancer genome Atlas. Cell.

[48] Cordes N., Blaese M.A., Meineke V., Van Beuningen D. (2002). Ionizing radiation induces up-regulation of functional β1-integrin in human lung tumour cell lines in vitro. Int J Radiat Biol.

[49] Eke I., Koch U., Hehlgans S., Sandfort V., Stanchi F., Zips D., et al. PINCH1 controls Akt1 for regulating radiation sensitivity by inhibiting PP1α Iris 2010;120.

[50] Stoeltzing O., Liu W., Reinmuth N., Fan F., Parry G.C., Parikh A.A. (2003). Inhibition of integrin α5β1 function with a small peptide (ATN-161) plus continuous 5-FU infusion reduces colorectal liver metastases and improves survival in mice. Int J Cancer.

[51] Lin L., Huang K., Tu Z., Zhu X., Li J., Lei K. (2021). Integrin Alpha-2 as a Potential Prognostic and Predictive Biomarker for Patients With Lower-Grade Glioma. Front Oncol.

[52] Deville S.S., Vehlow A., Förster S., Dickreuter E., Borgmann K., Cordes N. (2020). The intermediate filament synemin regulates non-homologous end joining in an ATM-dependent manner. Cancers (Basel).

[53] Dickreuter E., Eke I., Krause M., Borgmann K., Van Vugt M.A., Cordes N. (2016). Targeting of β1 integrins impairs DNA repair for radiosensitization of head and neck cancer cells. Oncogene.

[54] Jin S., Lee W.C., Aust D., Pilarsky C., Cordes N. (2019). β8 integrin mediates pancreatic cancer cell radiochemoresistance. Mol Cancer Res.

[55] Mandal C., Sarkar S., Chatterjee U., Schwartz-Albiez R., Mandal C. (2014). Disialoganglioside GD3-synthase over expression inhibits survival and angiogenesis of pancreatic cancer cells through cell cycle arrest at S-phase and disruption of integrin-β1-mediated anchorage. Int J Biochem Cell Biol.

[56] Sun C.C., Li S.J., Chen Z.L., Li G., Zhang Q., Li D.J. (2018). Expression and prognosis analyses of runt-related transcription factor family in human leukemia. Mol Ther Oncolytics.

[57] Wang N., Zhu L., Xu X., Yu C., Huang X. (2022). Integrated analysis and validation reveal ACAP1 as a novel prognostic biomarker associated with tumor immunity in lung adenocarcinoma. Comput Struct Biotechnol J.

[58] Xu Y., Wang J., Li F., Zhang C., Zheng X., Cao Y. (2022). Identifying individualized risk subpathways reveals pan-cancer molecular classification based on multi-omics data. Comput Struct Biotechnol J.

[59] Görte J., Danen E., Cordes N. (2022). Therapy-Naive and Radioresistant 3-Dimensional Pancreatic Cancer Cell Cultures Are Effectively Radiosensitized by β1 Integrin Targeting. Int J Radiat Oncol Biol Phys.

[60] Koppenhagen P., Dickreuter E., Cordes N. (2017). Head and neck cancer cell radiosensitization upon dual targeting of c-Abl and beta1-integrin. Radio Oncol.

[61] Eke I., Deuse Y., Hehlgans S., Gurtner K., Krause M., Baumann M. (2012). β 1 Integrin/FAK/cortactin signaling is essential for human head and neck cancer resistance to radiotherapy. J Clin Invest.

[62] Kim Y.J., Jung K., Baek D.S., Hong S.S., Kim Y.S. (2017). Co-targeting of EGF receptor and neuropilin-1 overcomes cetuximab resistance in pancreatic ductal adenocarcinoma with integrin β1-driven Src-Akt bypass signaling. Oncogene.

[63] Bergonzini C., Kroese K., Zweemer A.J.M., Danen E.H.J. (2022). Targeting integrins for cancer therapy - disappointments and opportunities. Front Cell Dev Biol.

[64] Steglich A., Vehlow A., Eke I., Cordes N. (2015). Α integrin targeting for radiosensitization of three-dimensionally grown human head and neck squamous cell carcinoma cells. Cancer Lett.

[65] Hu T., Zhou R., Zhao Y., Wu G. (2016). Integrin α6/Akt/Erk signaling is essential for human breast cancer resistance to radiotherapy. Sci Rep.

[66] Kowalski-Chauvel A., Modesto A., Gouaze-andersson V., Baricault L., Gilhodes J., Delmas C. (2018). Alpha-6 integrin promotes radioresistance of glioblastoma by modulating DNA damage response and the transcription factor Zeb1. Cell Death Dis.

[67] Yang H., Xu Z., Peng Y., Wang J., Xiang Y. (2021). Integrin β4 as a Potential Diagnostic and Therapeutic Tumor Marker. Biomolecules.

[68] Lathia J.D., Gallagher J., Heddleston J.M., Wang J., Eyler C.E., MacSwords J. (2010). Integrin Alpha 6 Regulates Glioblastoma Stem Cells. Cell Stem Cell.

[69] Dobson H.E., Ruan S., Chang A.E., Wicha M.S., Li Q. (2021). Targeting cancer stem cells via integrin β4. Oncotarget.

[70] Kariya Y., Kariya Y. (2022). Osteopontin in cancer: mechanisms and therapeutic targets. Int J Transl Med.

[71] Corona A., Blobe G.C. (2021). The role of the extracellular matrix protein TGFBI in cancer. Cell Signal.

[72] Lee J., Lee J., Sim W., Kim J.H. (2022). Soluble TGFBI aggravates the malignancy of cholangiocarcinoma through activation of the ITGB1 dependent PPARγ signalling pathway. Cell Oncol.

[73] Han B., Cai H., Chen Y., Hu B., Luo H., Wu Y. (2015). The role of TGFBI (βig-H3) in gastrointestinal tract tumorigenesis. Mol Cancer.

[74] Vehlow A., Klapproth E., Jin S., Hannen R., Hauswald M., Bartsch J.W. (2019). Interaction of Discoidin Domain Receptor 1 with a 14-3-3-Beclin-1-Akt1 Complex Modulates Glioblastoma Therapy Sensitivity. Cell Rep.

[75] Rudra-Ganguly N., Lowe C., Mattie M., Chang M.S., Satpayev D., Verlinsky A. (2014). Discoidin Domain Receptor 1 Contributes to Tumorigenesis through Modulation of TGFBI Expression. PLoS One.

[76] Choi D.S., Choi D.Y., Hong B.S., Jang S.C., Kim D.K., Lee J. (2012). Quantitative proteomics of extracellular vesicles derived from human primary and metastatic colorectal cancer cells. J Extra Vesicles.

[77] Hurwitz S.N., Meckes D.G. (2019). Extracellular Vesicle Integrins Distinguish Unique Cancers. Proteomes.

[78] Uchihara T., Miyake K., Yonemura A., Komohara Y., Itoyama R., Koiwa M. (2020). Extracellular Vesicles from Cancer-Associated Fibroblasts Containing Annexin A6 Induces FAK-YAP Activation by Stabilizing β1 Integrin, Enhancing Drug Resistance. Cancer Res.

[79] Pietilä E.A., Gonzalez-Molina J., Moyano-Galceran L., Jamalzadeh S., Zhang K., Lehtinen L. (2021). Co-evolution of matrisome and adaptive adhesion dynamics drives ovarian cancer chemoresistance. Nat Commun.

[80] Naba A., Clauser K.R., Ding H., Whittaker C.A., Carr S.A., Hynes R.O. (2016). The extracellular matrix: Tools and insights for the “omics” era. Matrix Biol.

[81] Karagöz Z., Rijns L., Dankers P.Y.W., van Griensven M., Carlier A. (2020). Towards understanding the messengers of extracellular space: Computational models of outside-in integrin reaction networks. Comput Struct Biotechnol J.

