# Supplementary material for: Meta-analysis of expression and the targeting of cell adhesion associated genes in nine cancer types – A one research lab re-evaluation
Source: Comput Struct Biotechnol J. 2023 Apr 19;21:2824–36. doi: 10.1016/j.csbj.2023.04.017 (PMC10189096; doi:10.1016/j.csbj.2023.04.017)
Supplement: Supplementary file 1 — Supplementary material [file mmc1.docx]

**Supplementary Figures and Tables**

# Meta-analysis of expression and the targeting of cell adhesion associated genes in nine cancer types – A one research lab re-evaluation

Olegs Borodins ^a,1^, Felix Broghammer ^a,1^, Michael Seifert ^b,c^, Nils Cordes ^a,c,d,e,f,*^

^a^ OncoRay—National Center for Radiation Research in Oncology, Faculty of Medicine Carl Gustav Carus, Technische Universität Dresden, 01307 Dresden, Germany

^b^ Institute for Medical Informatics and Biometry (IMB), Faculty of Medicine Carl Gustav Carus, Technische Universität Dresden, 01307 Dresden, Germany

^c^ National Center for Tumor Diseases (NCT), Partner Site Dresden, German Cancer Research Center (DKFZ), 69192 Heidelberg, Germany

^d^ Helmholtz-Zentrum Dresden—Rossendorf (HZDR), Institute of Radiooncology—OncoRay, 01328 Dresden, Germany

^e^ German Cancer Consortium, Partner Site Dresden: German Cancer Research Center, 69120 Heidelberg, Germany

^f^ Department of Radiotherapy and Radiation Oncology, University Hospital Carl Gustav Carus, Technische Universität Dresden, 01307 Dresden, Germany

^1^ These authors contributed equally to this work.

^*^ **Corresponding Author:** Prof. Dr. Nils Cordes, Nils.Cordes@OncoRay.de

**Supplementary Figures**


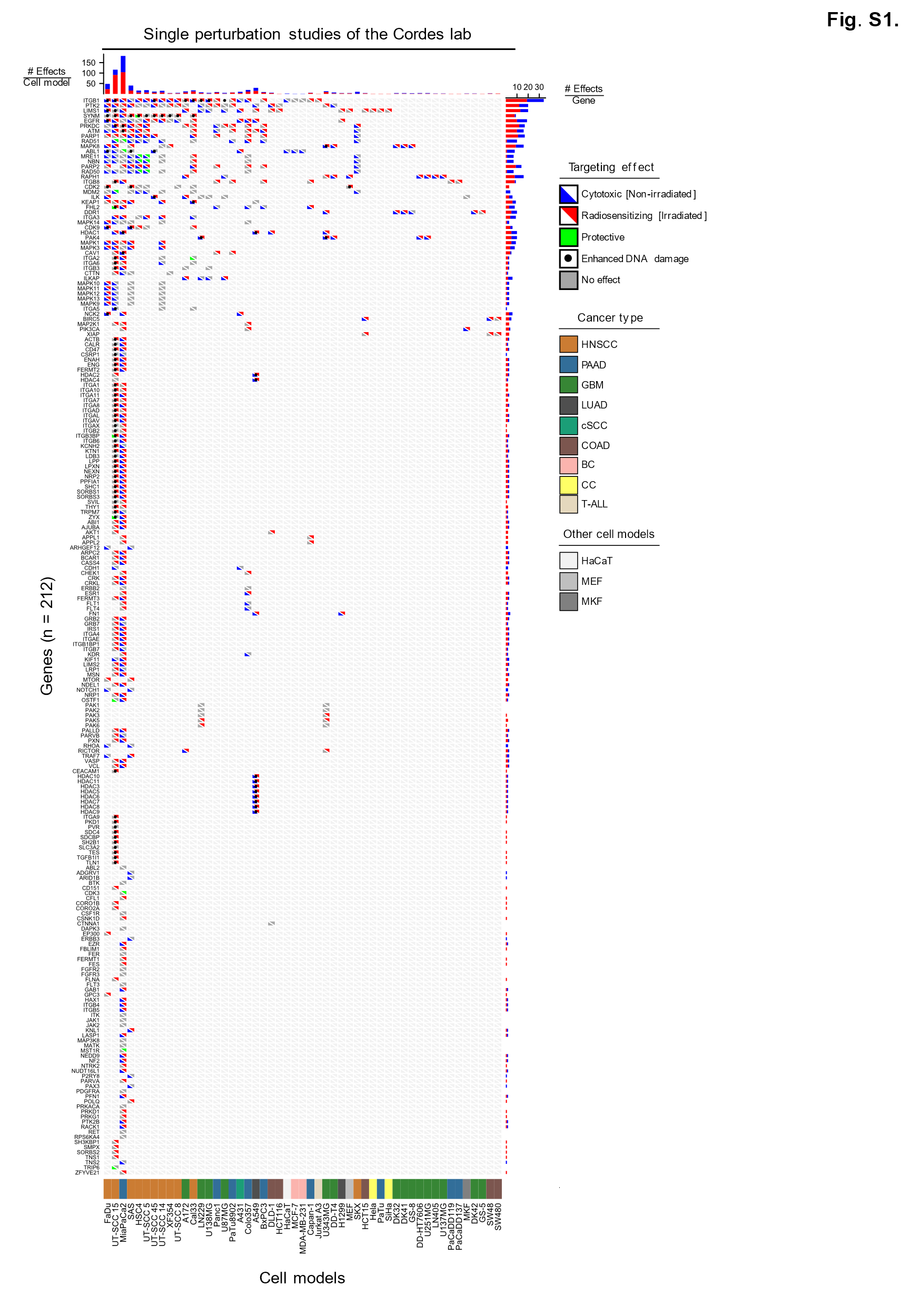


**Fig**. **S1**. Targeting effects in published Cordes lab cell models. Overview of individual targeted molecules investigated in the Cordes lab indicated by their gene name (n = 212) in rows and the 51 examined cell models in columns. Significant (p < 0.05) adverse effects on survival are designated as cytotoxic (blue; under non-irradiated conditions) and radiosensitizing (red; irradiation conditions). A significant increase in survival is designated as protective (green). A targeting-mediated enhancement in DNA damage after irradiation is indicated by black dots. Gray color indicates no effects on cell survival. HNSCC, Head and neck squamous cell carcinoma; PAAD, Pancreatic adenocarcinoma; GBM, Glioblastoma multiforme; LUAD, Lung adenocarcinoma; cSCC, Cutaneous squamous cell carcinoma; COAD, Colon adenocarcinoma; BC, Breast cancer; CC, Cervical cancer; T-ALL, T-cell acute lymphoblastic leukemia; HaCaT, Human keratinocytes; MEF, Mouse embryonic fibroblasts; MKF, Mouse kidney fibroblasts.





**Fig**. **S2**. PubMed literature search on top 30 investigated genes from the Cordes lab. Preclinical publications on these genes were evaluated for significant (p < 0.05) adverse effects (radiosensitization) in terms of cell survival (red) and DNA damage (black), or both (purple). In grey, publications including these genes but without targeting approaches and/or our selected adverse effects are shown. Recorded cytotoxic effects or DNA damage at basal conditions are provided in Table S1. (A) Strict literature search evaluating publications in which accurate targeting descriptions were provided in the text. (B) Literature search on radiation-specific publications identified by text-mining approaches.





**Fig**. **S3**. Nine heterogeneous cancer types display similar differential expression changes compared to the corresponding normal tissues. (A) Significantly (p < 0.05) differential expressed genes (DEGs) between cancer types (CTs) and corresponding normal tissues. (B) Principal component analysis of DEG fold changes of different CTs relative to normal tissues. (C) Similarly changed DEGs (scDEGs) from Figure 2B for each individual cancer type. Top 10 upregulated and downregulated genes are named. All statistical analyses considered p < 0.05 and FDR < 0.05. LUSC, Lung squamous cell carcinoma; LUAD, Lung adenocarcinoma; PRAD, Prostate adenocarcinoma; COAD, Colon adenocarcinoma; READ, Rectum adenocarcinoma; LGG, Lower grade glioma; GBM, Glioblastoma multiforme; HNSCC, Head and neck squamous cell carcinoma; PAAD, Pancreatic adenocarcinoma.





**Fig**. **S4**. Non-similarly changed DEGs discriminate the cancer types functionally. (A-D) Differentially expressed genes between cancer versus normal tissue were evaluated for similar expression changes. All genes which did not pass our similar expression criteria were named non-similarly changed DEGs (non-scDEGs) and subjected to functional enrichment analysis. Each analysis is based on different databases: (A) GO biological processes, (B) KEGG pathways, (C) GO complete database, and (D) Reactome pathways. All terms are ranked by gene ratio enrichment scores indicated by symbol size. The color coding indicates the FDR significance level (all FDR < 0.05). COAD, Colon adenocarcinoma; GBM, Glioblastoma multiforme; HNSCC, Head and neck squamous cell carcinoma; LGG, Lower grade glioma; LUAD, Lung adenocarcinoma; LUSC, Lung squamous cell carcinoma; PAAD, Pancreatic adenocarcinoma; PRAD, Prostate adenocarcinoma; READ, Rectum adenocarcinoma.



**Fig**. **S5**. K-Mean / PCA clustering details. Combined K-mean / PCA data analysis of adhesion associated scDEGs and corresponding reciprocal p-values for TCGA patient survival. Data sets were grouped into positive and negative categories according to their impact on “All” (A) and “RT” (B) survival. The plots describe: K-mean cluster identification on PCA coordinates (top left); individual points of the cancer type data distribution (top right); adhesion associated scDEGs classification based on up- or downregulated expression (middle left); and positive or negative impact on patient OS (middle right); and localization of up- and downregulated genes with respective positive or negative impacts on survival (bottom left). Overexpressed genes with a negative impact on OS in a minimum of two cancer types were selected as candidates for further selection (bottom right; aaGOI candidates). Adhesion associated genes of interest, aaGOIs; Overall survival, OS; LUSC, Lung squamous cell carcinoma; LUAD, Lung adenocarcinoma; PRAD, Prostate adenocarcinoma; COAD, Colon adenocarcinoma; READ, Rectum adenocarcinoma; LGG, Lower grade glioma; GBM, Glioblastoma multiforme; HNSCC, Head and neck squamous cell carcinoma; PAAD, Pancreatic adenocarcinoma.





**Fig**. **S6**. TCGA survival analyses of adhesion associated genes of interest. The heatmaps depict the pancancer survival analyses from Figure 3C split into the individual TCGA cancer type cohorts. COAD and READ patients were combined. For each gene, the patients of the respective TCGA studies were divided in high and low gene expression cohorts (group cutoff: median expression) and subjected to Kaplan-Maier analysis. Heatmaps highlight the impact of the overexpressed aaGOIs on patient OS by log-rank test significance cutoffs (p < 0.1, 0.05, 0.1). The assigned target gene group from Figure 3C, describing which patient (sub-)population was most affected, is annotated to the right of each heatmap. Source data is provided in Table S3. Adhesion associated genes of interest, aaGOIs; OS, overall survival; LUSC, Lung squamous cell carcinoma; LUAD, Lung adenocarcinoma; PRAD, Prostate adenocarcinoma; COAD, Colon adenocarcinoma; READ, Rectum adenocarcinoma; LGG, Lower grade glioma; GBM, Glioblastoma multiforme; HNSCC, Head and neck squamous cell carcinoma; PAAD, Pancreatic adenocarcinoma.

**Supplementary Tables**

**Table S1**. Experimental data on Cordes lab genes (see Excel file).

**Table S2**. Similarly changed DEGs in cancer types and cell models (see Excel file).

**Table S3**. TCGA survival analyses data (see Excel file).

**Table S4**. Functional enrichment analyses (see Excel file).
